# Editorial: Contribution of omics to our understanding of human-bacterial interplay in health and disease

**DOI:** 10.3389/fgene.2023.1172822

**Published:** 2023-03-13

**Authors:** Nicolas Nalpas, Tariq Ganief, Julie Hardouin

**Affiliations:** ^1^ Department of Biology, Quantitative Proteomics Group, Interfaculty Institute of Cell Biology, University of Tübingen, Tübingen, Germany; ^2^ Division of Chemical & Systems Biology, Department of Integrative Biomedical Sciences, Faculty of Health Sciences, University of Cape Town, Cape Town, South Africa; ^3^ University of Rouen Normandie, INSA Rouen Normandie, CNRS, Polymers, Biopolymers, Surfaces Laboratory UMR 6270, Rouen, France; ^4^ University of Rouen Normandie, INSERM US 51, CNRS UAR 2026, HeRacLeS-PISSARO, Normandie Université, Rouen, France

**Keywords:** microbiome, bacterial pathogen, proteomic, genomic, transcriptomic

Since their discovery by Antoni van Leeuwenhoek in 1676, bacterial microorganisms have not ceased to amaze by the impact they can have on us. Recent estimates bring their census to nearly one trillion species (Locey and Lennon, 2016) with genomic diversity that far surpasses the combined diversity of archaea and eukaryotes (Hug et al., 2016). Indeed, bacterial organisms have evolved on our planet for over 3.5 billion years and have adapted to even the most extreme environments (e.g., deep sea trenches, polar regions, hyperacidic lakes, deserts) (Merino et al., 2019). In this time, they have developed phenomenal adaptations to survive, including the ability to promote their survival within a host as parasites and commensals. Consequently, our health depends on the many bacteria that make up our various microbiomes, but also on pathogenic or opportunistic bacteria. Their epidemiological, socio-economic and politico-historical importance has only been truly appreciated during the last 150 years, for example, with the ongoing development of bacterial multi-drug resistance, or for the benefits provided by a healthy microbiota. In the past 30 years, omic approaches have greatly enhanced our understanding of biological systems (Veenstra, 2021). However, there is still a lack of literature on bacterial organisms despite their significance (i.e., pathogenic bacteria, host-pathogen interactions, and microbiota). Thus, this research topic aimed to highlight the role of omics in comprehending bacterial systems and their interactions with humans.

In this context, Holmes et al. discussed the transmission dynamics of a hypervirulent *Neisseria meningitidis* serogroup W ST-11 clone in a student population with an 11-fold increase in carriage. The study used whole-genome sequencing and bioinformatic analysis to identify clusters associated with residential halls and determine transmission rates. Effective contacts and immunity levels were found to be important factors in the spread of the disease, while genomic variability was not as significant. Super-spreaders were also found to enhance transmission and a 70% immunization level with Meningitis ACWY vaccine was deemed sufficient to retard, but not fully prevent, meningococcal spread in close-contact populations. Balamurugan et al. studied the genetic diversity of *Mycobacterium tuberculosis* var. *africanum* (Maf) in West Africa using whole-genome sequencing and single nucleotide polymorphisms (SNPs). The study identified two lineages of Maf, L5 and L6, using region of difference (RD) based methods, and it used SNPs to identify sub-lineages within these. Specific SNPs were identified as biomarkers and used for validation. The study suggests that cluster-specific SNPs could be used as additional markers for Maf delineation and could provide insights into the genotype-phenotype correlation and endemicity of Maf in the African population. Liu et al. investigated target genes against *Streptococcus gordonii* that are found in *S. mutans*, a pathogen responsible for dental caries. The study used 80 clinical isolates of *S. mutans* and identified 33 genes positively correlated and 61 genes negatively correlated with *S. mutans* against *S. gordonii*. RNA-sequencing and qRT-PCR were used to identify critical gene clusters against *S. gordonii* in *S. mutans* clinical isolates. In the study of Hardouin et al., the microbial components of spontaneous and induced sputum samples from three cystic fibrosis (CF) patients were compared using tandem mass spectrometry-based proteotyping. The study found no significant difference in microorganism abundance between paired spontaneous and induced sputum samples and observed microbial proteins linked to resistance, iron uptake, and biofilm-forming ability in sputa. This method could be highly complementary to culture for clinical management of CF patients, improving knowledge about the host-pathogen dynamics and CF pathophysiology. Zhang et al. reported on a multidrug-resistant strain of *Stenotrophomonas acidaminiphila*, a relatively rare bacteria, isolated from a sepsis patient. The genome of the strain was sequenced and compared with six closely related strains, revealing strain-level diversity and identifying unique genes important for pathogenesis. The strain was found to be resistant to several antibiotics, with specific antibiotic resistance genes responsible for multidrug resistance. The study provides insight into the relationship between the strain’s phenotype and genotype, and potential therapeutic strategies for infections.

In this Research Topic, a large diversity of bacterial organisms were studied. These were isolated from different colonisation sites (e.g., dental, sputum, brain) where they caused a variety of pathologies (e.g., sepsis, meningitis, dental caries). The use of genomics, transcriptomics, or proteomics approaches allowed the interrogation of these complex systems. The authors were able to identify biomarkers of pathogenicity or antibiotic-resistance, as well as identify genes of interest during bacterial competition. Beyond these specific conclusions, the data generated within these studies will feed into the pool of scientific knowledge and contribute to a larger understanding of bacteria.

## References

[B1] Hug L. A. Baker B. J. Anantharaman K. Brown C. T. Probst A. J. Castelle C. J. (2016). A new view of the tree of life. Nat. Microbiol. 1, 16048. 10.1038/nmicrobiol.2016.48 27572647

[B2] Locey K. J. Lennon J. T. (2016). Scaling laws predict global microbial diversity. Proc. Natl. Acad. Sci. 113, 5970–5975. 10.1073/pnas.1521291113 27140646 PMC4889364

[B3] Merino N. Aronson H. S. Bojanova D. P. Feyhl-Buska J. Wong M. L. Zhang S. (2019). Living at the extremes: Extremophiles and the limits of life in a planetary context. Front. Microbiol. 10, 780. 10.3389/fmicb.2019.00780 31037068 PMC6476344

[B4] Veenstra T. D. (2021). Omics in systems biology: Current progress and future outlook. PROTEOMICS 21, 2000235. 10.1002/pmic.202000235 33320441

